# Regulation of pyroptosis by natural products in atherosclerosis: mechanisms and therapeutic potential

**DOI:** 10.3389/fphar.2025.1626566

**Published:** 2025-09-04

**Authors:** Na Shi, Lai Wei, He Wang, Shude Sun, Jianfei Yang, Yabin Zhou

**Affiliations:** ^1^ Graduate School, Heilongjiang University of Chinese Medicine, Harbin, Heilongjiang, China; ^2^ Department of Cardiovascular Medicine, First Affiliated Hospital of Heilongjiang University of Chinese Medicine, Harbin, Heilongjiang, China

**Keywords:** atherosclerosis, pyroptosis, mechanisms, natural products, Traditional Chinese Medicine

## Abstract

Pyroptosis, a proinflammatory form of programmed cell death characterized by inflammasome activation and gasdermin-mediated membrane pore formation, has been recognized as a critical contributor to the initiation and progression of atherosclerosis (AS). Increasing evidence indicates that pyroptosis accelerates plaque formation and rupture by promoting endothelial dysfunction, vascular smooth muscle cell loss, and destabilization of macrophage-derived foam cells. Given its pivotal role in AS pathogenesis, modulation of pyroptotic signaling pathways is considered a highly promising therapeutic strategy. Natural products derived from medicinal plants and dietary sources possess multiple biological activities, including antioxidant, anti-inflammatory, and lipid-regulating effects, and their potential to target pyroptosis in cardiovascular diseases has attracted growing attention in recent years. This review systematically summarizes current advances in understanding the regulatory effects of natural products on pyroptosis in AS. Representative compounds, including polyphenols, flavonoids, saponins, and alkaloids, have been shown in AS animal models to inhibit inflammasome assembly, block gasdermin cleavage, and restore vascular homeostasis. This review focuses on elucidating the mechanisms and therapeutic potential of natural products in regulating pyroptosis in AS, aiming to provide a reference for their application in AS treatment and to lay a foundation for the development of effective new drugs for AS prevention and management.

## 1 Introduction

Cardiovascular disease (CVD) remains the leading cause of death and disability worldwide, with AS serving as its principal pathological basis (Flora and Nayak, 2019; Vaduganathan et al., 2022). AS is a multifactorial, chronic inflammatory disorder characterized by lipid metabolism dysregulation and immune activation. It is defined by the accumulation of lipids in the arterial intima, accompanied by vascular smooth muscle cell (VSMC) migration and abnormal proliferation of the extracellular matrix, ultimately leading to plaque formation, luminal narrowing, and impaired blood flow (Libby and Hansson, 2015). The development of AS is influenced by several risk factors, including hyperlipidemia, smoking, hypertension, and diabetes. Its pathogenesis is driven by complex interactions among endothelial cells (ECs), VSMCs, macrophages, and other immune cells, coupled with chronic inflammation and progressive lipid deposition within the arterial wall (Libby, 2021). In the early stages of AS, hypercholesterolemia and hemodynamic disturbances stimulate ECs to upregulate adhesion molecules and increase vascular permeability, thereby promoting the subendothelial infiltration of monocytes and low-density lipoproteins (LDL) (Grootaert and Bennett, 2021). These monocytes differentiate into macrophages within the intima and internalize lipids to become foam cells (Bu et al., 2023). As the disease advances, foam cell formation by both macrophages and VSMCs, along with extracellular matrix remodeling, leads to the development of atheromatous plaques (Tabas and Bornfeldt, 2016). In late-stage AS, the accumulation of necrotic cells, cell debris, and cholesterol crystals contributes to the formation of a necrotic core, destabilizing the plaque and markedly increasing the risk of thrombosis (Grootaert et al., 2015; Qian et al., 2021). Consequently, cell death and its associated inflammatory processes are recognized as central contributors to the pathophysiology of AS.

Currently, therapeutic strategies for AS predominantly aim to lower lipid levels, with statins being the most widely prescribed agents. However, prolonged use of statins may lead to adverse effects and fails to fully suppress the inflammation-driven progression of atherosclerotic lesions. This limitation underscores the urgent need for novel therapeutic approaches that can more effectively address both the metabolic and inflammatory components of AS. In recent years, pyroptosis—a distinct form of programmed cell death—has garnered increasing attention for its pivotal role in inflammatory and cardiovascular diseases (Zhaolin et al., 2019; Lin et al., 2022; Vasudevan et al., 2023). Pyroptosis is a caspase-dependent, pro-inflammatory mode of cell death, characterized by membrane pore formation, release of pro-inflammatory cytokines, and subsequent cell lysis (Shi et al., 2017). Pyroptosis not only represents a regulated form of cell death but also functions as a potent amplifier of inflammatory responses (Lin et al., 2021; Li M. et al., 2022). Pyroptotic cells release pro-inflammatory cytokines such as interleukin-1β (IL-1β) and interleukin-18 (IL-18), which exacerbate the local inflammatory milieu and accelerate disease progression (Yu J. et al., 2021; Ding et al., 2024). Inflammation serves both as an initiating factor and a driving force in the pathogenesis of AS (Newton et al., 2021). Accumulation and dysfunction of macrophages are key pathological features of AS, and pyroptosis activation in these cells promotes the release of inflammatory mediators and induces secondary necrosis, facilitating the formation of a necrotic core and compromising plaque stability (De Meyer et al., 2024). Moreover, pyroptosis in ECs and VSMCs has been closely associated with vascular dysfunction and thinning of the fibrous cap, further aggravating the atherosclerotic process (Qian et al., 2021). These findings collectively underscore the multifaceted roles of pyroptosis in AS development and progression.

Although growing evidence supports the involvement of pyroptosis in the pathogenesis of AS, its underlying regulatory mechanisms remain incompletely elucidated. Key uncertainties persist regarding the temporal dynamics of pyroptosis, cell type-specific pyroptotic patterns at various stages of AS progression, and the interplay among different pyroptotic pathways within vascular tissues (Jia et al., 2019; Toldo and Abbate, 2024). Moreover, whether therapeutic modulation of pyroptosis signaling can effectively mitigate AS progression requires further validation through preclinical and clinical studies. Natural products offers distinct advantages in AS management due to its multi-target and multi-pathway actions, along with a favorable safety profile. Increasing research has demonstrated that natural products can regulate pyroptosis and confer protective effects against atherosclerotic lesions (Song and Chen, 2021; Zheng Y. et al., 2022). Against this backdrop, the paper focuses on pyroptosis as a key entry point to systematically explore its molecular mechanisms, signaling pathways, and potential therapeutic targets in AS. It also reviews the research progress on the role of natural products in preventing and treating AS through the regulation of pyroptosis.

## 2 Review methodology

To explore the mechanisms by which natural products interventions exert therapeutic effects on AS through the regulation of pyroptosis, we conducted a comprehensive literature search across multiple databases, including Google Scholar, Medline, PubMed, Scopus, and the China National Knowledge Infrastructure (CNKI). The search strategy integrated controlled vocabulary and free-text keywords with Boolean operators, focusing on four conceptual clusters: (1) AS-related terms (“atherosclerosis” OR “AS”), (2) pyroptosis (“pyroptosis” OR “gasdermin D” OR “caspase-1”), (3) NLRP3 (“NLRP3” OR “NOD-like receptor protein 3”), and (4) therapeutic interventions (“natural products” OR “Traditional Chinese Medicine (TCM)” OR “herb”). All retrieved articles were independently screened by two reviewers based on their titles, abstracts, and full texts, in accordance with predefined inclusion and exclusion criteria. Inclusion criteria: (1) Original research articles; (2) Studies investigating the effects of natural products on pyroptosis-related pathways in AS models. Exclusion criteria: (1) Gray literature; (2) Editorials, commentaries, and review articles; (3) Duplicate publications.

## 3 Molecular mechanisms of pyroptosis

Cell death is an essential physiological process involved in development, homeostasis, and responses to injury (Liu et al., 2024). Based on its regulatory mechanisms, cell death can be classified into accidental cell death (ACD) and regulated cell death (RCD) (Galluzzi et al., 2018). RCD is a genetically encoded and tightly controlled process, often occurring as part of cellular senescence or adaptive responses to internal or external stressors (Galluzzi et al., 2018). Various forms of RCD have been identified, including apoptosis, necroptosis, pyroptosis, and ferroptosis (Green, 2019).

Pyroptosis is a form of programmed cell death mediated by inflammasomes, and is characterized by membrane rupture, cell swelling, the release of pro-inflammatory cytokines such as IL-1β and IL-18, and a robust inflammatory response (Chen et al., 2016; Xu et al., 2019; Bertheloot et al., 2021). Its lytic nature and pronounced pro-inflammatory effects distinguish pyroptosis from other types of RCD, such as necroptosis and ferroptosis (Bertheloot et al., 2021; Qin J. et al., 2024). The concept of pyroptosis dates back to 1986, when Friedlander first reported that anthrax toxin could induce macrophage lysis (Fink and Cookson, 2005). In 1992, Zychlinsky and colleagues demonstrated that *Shigella flexneri* infection triggered rapid lysis of macrophages through a pathway independent of caspase-3 and Bcl-2 regulation (Zychlinsky et al., 1992). It was not until 2001 that Cookson and colleagues formally coined the term “pyroptosis” to describe this unique form of inflammatory cell death. Derived from the Greek words *pyro* (fire) and *ptosis* (falling), the term reflects the intense inflammatory response associated with this process (Cookson and Brennan, 2001). Subsequent studies have established that pyroptosis is mediated by caspase-1, caspase-4, caspase-5, and the murine homolog caspase-11, marking it as a distinct form of regulated cell death (Shi et al., 2014). In 2015, Kayagaki et al. and Shi et al. independently identified gasdermin D (GSDMD) as the key executioner protein of pyroptosis. Upon cleavage by activated caspases, the N-terminal domain of GSDMD translocates to the plasma membrane, where it forms pores that allow water influx, leading to cell swelling and membrane rupture (Kayagaki et al., 2015; Shi et al., 2015). In 2018, the Nomenclature Committee on Cell Death (NCCD) formally defined pyroptosis as a form of programmed cell death that is dependent on gasdermin-mediated plasma membrane permeabilization (Galluzzi et al., 2018).

The central mechanism of pyroptosis is driven by inflammasome activation, which initiates the cleavage of gasdermin (GSDM) family proteins by caspase enzymes. This process results in plasma membrane rupture and the release of pro-inflammatory cytokines, playing dual roles in host immune defense and pathological tissue injury (He et al., 2015; Shi et al., 2015; Ding et al., 2016). GSDM, a key member of the GSDM superfamily, belongs to a group of pore-forming proteins exclusively identified in vertebrates. This superfamily includes human gasdermins A through E and DFNB59 (also known as Pejvakin, PJVK), as well as murine homologs such as Gsdma1/2/3, Gsdmc1/2/3/4, Gsdmd, Dfna5, and Dfnb59 (Kuang et al., 2017; Liu et al., 2019). Except for DFNB59, all members of the gasdermin family possess pore-forming capabilities and serve as critical effectors of pyroptosis (Ding et al., 2016). The N-terminal domain (NT) is the functional fragment responsible for membrane pore formation, while the C-terminal domain (CT) acts as an autoinhibitory module under resting conditions, preventing unintended activation of pyroptosis (Rogers et al., 2019). Upon appropriate stimuli, various proteases cleave specific GSDM proteins, releasing the NT domain. The active NT fragment then binds to phospholipids in the plasma membrane, mitochondria, or other organelles, forming transmembrane pores that trigger cell swelling, lysis, and the release of inflammatory mediators (Liu et al., 2016). As such, gasdermins are widely recognized as the executioners of pyroptosis (Shi et al., 2015).

**FIGURE 1:**
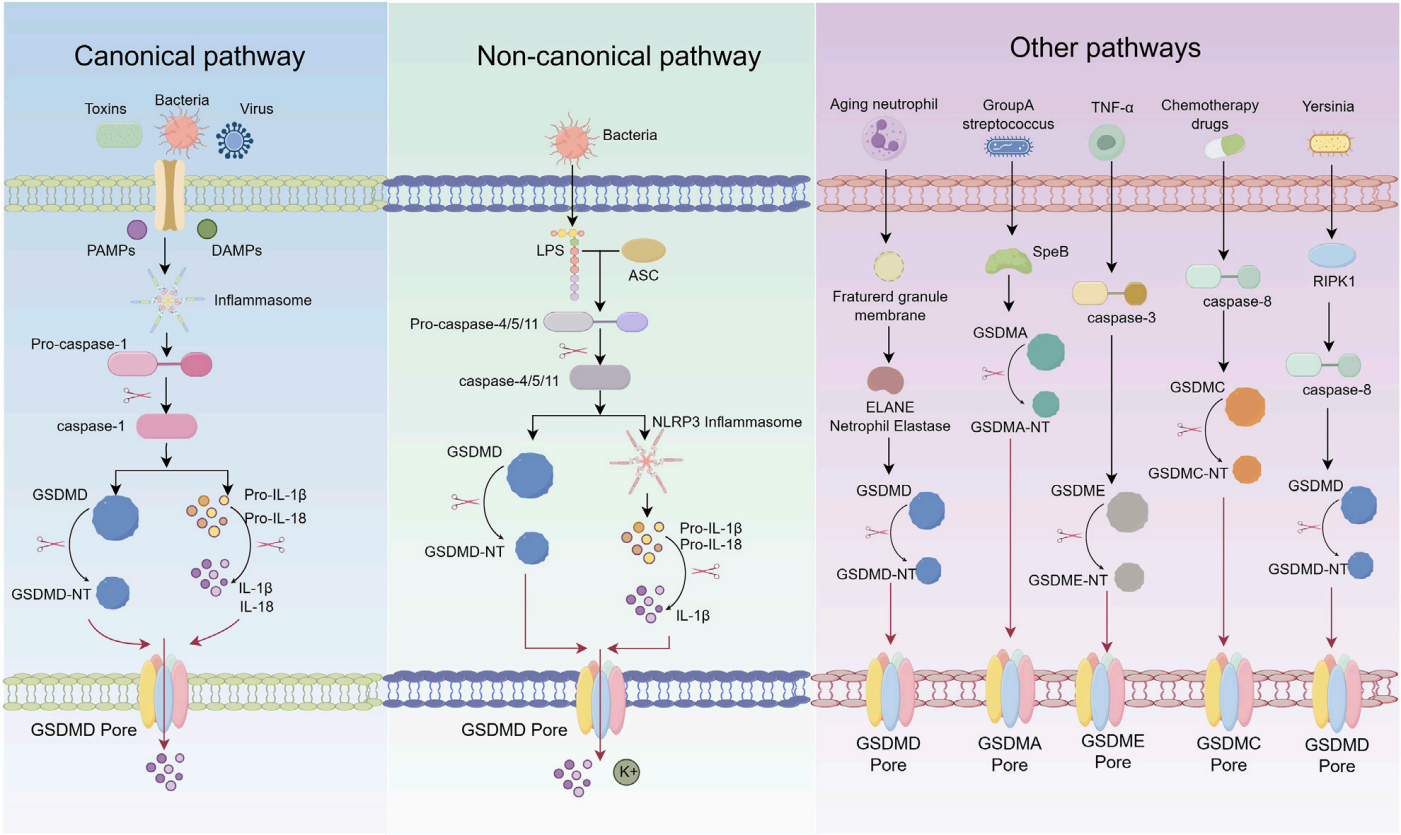
Schematic representation of pyroptosis pathways.

Pyroptosis should be regarded not merely as a distinct form of programmed cell death, but as a pivotal regulatory node within the host immune stress response network. Despite substantial progress in elucidating its basic pathways, critical aspects of its molecular mechanisms remain unresolved. In particular, the marked heterogeneity in pyroptotic signaling observed across different cell types and under varying pathological stimuli suggests a high degree of context-dependent specificity and dynamic regulation. Accordingly, this review seeks to systematically delineate the signaling mechanisms and regulatory frameworks of pyroptosis—encompassing canonical, non-canonical, and other emerging pathways—with the aim of providing a comprehensive theoretical foundation for understanding its pathogenic roles in AS. The specific details of the pyroptosis process are shown in Figure 1.

### 3.1 Canonical pyroptosis pathway

The canonical pyroptosis pathway is typically initiated by the recognition of pathogen-associated molecular patterns (PAMPs) or damage-associated molecular patterns (DAMPs) by pattern recognition receptors (PRRs) (He and Amer, 2014). This recognition activates inflammasome complexes—such as NLRP3, NLRP1, AIM2, and NLRC4—which in turn trigger the activation of caspase-1. Activated caspase-1 cleaves the precursors of interleukin-1β (pro-IL-1β) and interleukin-18 (pro-IL-18) into their mature, biologically active forms. It also cleaves GSDMD, resulting in pore formation in the plasma membrane and subsequent release of intracellular contents (Barnett et al., 2023; Zhang et al., 2024a). Upon detection of infectious or injury-related signals, PRRs specifically activate corresponding inflammasome sensors, such as members of the NOD-like receptor (NLR) family or AIM2 (Liston and Masters, 2017). These receptors interact with the adaptor protein apoptosis-associated speck-like protein containing a CARD (ASC) through pyrin domains (PYD) or caspase recruitment domains (CARD), facilitating the assembly of the inflammasome complex (Lin et al., 2024). ASC then recruits pro-caspase-1 via CARD–CARD interactions, enabling its proximity-induced autocatalytic activation into the mature, enzymatically active form (Broz and Dixit, 2016).

Caspase-1 plays dual roles in pyroptosis execution (Liu et al., 2016). First, it cleaves GSDMD, liberating its N-terminal fragment (GSDMD-NT), which oligomerizes and embeds into the plasma membrane to form transmembrane pores. These pores disrupt ionic homeostasis, leading to cell swelling, membrane rupture, and the onset of pyroptosis. Second, caspase-1 processes pro-IL-1β and pro-IL-18 into their active cytokine forms, which are released through GSDMD pores, thereby amplifying local inflammation, recruiting immune cells, and enhancing host defense responses (Shi et al., 2015).

The activation of inflammasomes is subject to multiple regulatory mechanisms (de Zoete et al., 2014). For example, NLRP3 activation is often dependent on cathepsins released from ruptured lysosomes and the accumulation of reactive oxygen species (ROS) generated by mitochondrial dysfunction (Zheng D. et al., 2022). NIMA-related kinase 7 (NEK7) has been identified as a critical regulator of NLRP3 inflammasome activation (Shi et al., 2016). Moreover, different inflammasomes are selectively activated by specific PAMPs and DAMPs: NLRC4 responds primarily to components of the type III secretion system (T3SS) in Gram-negative bacteria, while AIM2 specifically recognizes cytosolic double-stranded DNA (dsDNA) (Hornung et al., 2009; Kofoed and Vance, 2011). Notably, the membrane protein Ninjurin-1 (NINJ1) facilitates the terminal stage of pyroptosis by promoting plasma membrane rupture via oligomerization, further enhancing the execution of cell death (Kayagaki et al., 2019).

### 3.2 Non-canonical pyroptosis pathway

Unlike the canonical pyroptosis pathway, which is dependent on caspase-1 and inflammasome activation, the non-canonical pathway is primarily mediated by caspase-11 in mice and caspase-4/5 in humans, and functions independently of inflammasome assembly (Shi et al., 2014; Jiang et al., 2021). This pathway is activated by lipopolysaccharide (LPS), a component of Gram-negative bacterial cell walls, which directly binds to the N-terminal CARD of caspase-4/5/11, inducing their autoactivation (Ma et al., 2021).

Once activated, caspase-4/5/11 cleave GSDMD, releasing its GSDMD-NT (Shi et al., 2015). This fragment integrates into the plasma membrane to form transmembrane pores, disrupting ionic homeostasis, inducing cellular swelling and lysis, and leading to the release of intracellular contents and inflammatory mediators (Zeng et al., 2022). Although caspase-4/5/11 do not directly process pro-IL-1β and pro-IL-18, GSDMD-mediated pore formation facilitates potassium (K^+^) efflux, which subsequently activates the NLRP3 inflammasome. This, in turn, triggers caspase-1-dependent maturation and secretion of IL-1β and IL-18, thereby amplifying the inflammatory response (Rühl and Broz, 2015). Thus, the non-canonical pathway not only initiates pyroptosis but also indirectly promotes inflammation via NLRP3 activation. Additional regulatory elements in the non-canonical pathway include pannexin-1 and the purinergic receptor P2X7. Caspase-11 cleaves pannexin-1, promoting ATP release, which activates P2X7 signaling, further enhancing K^+^ efflux and NLRP3 inflammasome activation (Zhou et al., 2021). Moreover, NLRP3 has been shown to modulate GSDME expression and mediate pyroptosis in T cells, suggesting that the non-canonical pathway may have broader roles in immune regulation beyond its classical functions (Rogers et al., 2019).

### 3.3 Other pathways

Recent studies have uncovered multiple non-canonical mechanisms of pyroptosis beyond the classical caspase-1/GSDMD axis. Caspase-3, traditionally associated with apoptosis and considered inert toward gasdermin proteins, has now been shown to cleave GSDME. The resulting GSDME-NT forms membrane pores and triggers pyroptosis (Wang et al., 2017). Caspase-8 has also been reported to cleave both GSDME and GSDMD. Under inflammatory stimulation, caspase-8 can interact with programmed death-ligand 1 (PD-L1) to activate GSDMC, initiating a non-canonical pyroptotic pathway. This mechanism is particularly evident under hypoxic conditions, where signal transducer and activator of transcription 3 (STAT3) promotes GSDMC transcription and enhances caspase-8–dependent pyroptosis in the presence of tumor necrosis factor-α (TNF-α) (Zhou Z. et al., 2020). Recent studies have demonstrated that during *Yersinia* pseudotuberculosis infection, the caspase-8-mediated activation of GSDMD represents a key mechanism driving pyroptosis. Notably, the activity of this pathway is significantly regulated by the cellular metabolic state, particularly glucose availability. A decrease in glucose levels activates the intracellular energy sensor AMPK, which subsequently phosphorylates RIPK1 at the Ser321 site, thereby inhibiting its activation and ultimately suppressing downstream GSDMD-mediated pyroptosis (Yang et al., 2024).

Pyroptosis is not invariably dependent on caspase activation. In certain specialized cell types, such as senescent neutrophils, pyroptosis can be triggered independently of inflammasome signaling. This occurs through the cleavage of GSDMD by the neutrophil-specific serine protease elastase (ELANE), bypassing the canonical caspase-1 pathway (Kambara et al., 2018). Similarly, in human epidermoid carcinoma A431 cells infected with Group A *Streptococcus* (GAS), the bacterial protease SpeB directly cleaves GSDMA, releasing its GSDMA-NT, which initiates pyroptotic cell death (Deng et al., 2022).

Emerging evidence highlights the role of cytotoxic lymphocytes in pyroptosis induction. Chimeric antigen receptor (CAR) T cells have been shown to activate caspase-3 via the release of granzyme B (GzmB), subsequently triggering GSDME-dependent pyroptosis and widespread cell death (Liu Y. et al., 2020). Additionally, natural killer cells and cytotoxic T lymphocytes utilize granzyme A (GzmA) to cleave GSDMB at Lys229 and Lys244, thereby promoting pyroptosis in GSDMB-positive cells (Zhou Z. et al., 2020). Notably, GzmA can cleave gasdermin family members at non-aspartic acid residues, leading to pore formation and challenging the traditional paradigm of caspase-dependent pyroptosis. The function of GSDMB also varies among its splice variants; only specific isoforms generate N-terminal fragments capable of inducing pyroptosis upon proteolytic cleavage (Zhou Z. et al., 2020; Zhong et al., 2023).

Ninj1 has recently been identified as a critical executor of membrane rupture during the terminal phase of pyroptosis. Following GSDMD pore formation, calcium influx activates Ninj1, promoting its phosphorylation and oligomerization. This cooperates with gasdermin-mediated pores to disrupt the plasma membrane, thereby enhancing cell lysis and the release of inflammatory mediators (Kayagaki et al., 2021; Degen et al., 2023). In addition to Ninj1, the activation and membrane targeting of GSDMD are tightly regulated by post-translational modifications. Specifically, GSDMD undergoes reversible S-palmitoylation at cysteine 192, which facilitates its cleavage by caspases via DHHC7-mediated palmitoylation. Following cleavage, APT2 removes the palmitoyl group to expose C192, promoting oligomerization and membrane localization; disruption of this process significantly suppresses pyroptosis (Zhang et al., 2024b). Moreover, GSDMD is also subject to other modifications such as ubiquitination, phosphorylation, and oxidation, which collectively contribute to a dynamic regulatory network that modulates pyroptosis under various pathological conditions (Zhang and Xu, 2025).

MicroRNAs (miRNAs) have emerged as important regulators of pyroptosis, modulating the expression of critical molecules such as NLRP3 and GSDMD. For example, miR-223 negatively regulates NLRP3 mRNA, thereby reducing inflammasome assembly, caspase-1 activation, and GSDMD cleavage, ultimately alleviating plaque inflammation in AS. Similarly, miR-30e targets GSDMD and inhibits the formation of pyroptotic pores, attenuating cell death (Zhang et al., 2018; Song et al., 2022).

Recent advances in cell death research have identified PANoptosis as a novel, integrated form of regulated cell death. PANoptosis involves the crosstalk and co-regulation of pyroptosis, apoptosis, and necroptosis, orchestrated by a multiprotein complex termed the PANoptosome (Xiang et al., 2024). Key sensors such as NLRP3 and Z-DNA binding protein 1 (ZBP1) can simultaneously activate caspase-1, caspase-8, and RIPK3, thereby initiating diverse cell death pathways (Wang and Kanneganti, 2021). In the context of AS, oxidized low-density lipoprotein (oxLDL) has been shown to promote PANoptosome formation, driving inflammatory responses and foam cell death, which further contribute to the destabilization of atherosclerotic plaques (Bae et al., 2024).

## 4 The relationship between pyroptosis and AS

AS, a representative chronic inflammatory cardiovascular disease, progresses through a series of interrelated pathological events, including endothelial dysfunction and rupture, lipid accumulation and foam cell formation, recruitment and activation of immune cells—particularly monocyte-derived macrophages—and phenotypic switching of VSMCs, leading to fibrous cap destabilization. Increasing evidence indicates that pyroptosis in ECs, macrophages, and VSMCs plays a pivotal role in each of these stages. Pyroptotic ECs disrupt the vascular barrier, promote lipid infiltration and leukocyte adhesion, initiating atherogenesis. Macrophage pyroptosis enhances cytokine release and necrotic core expansion, accelerating plaque progression. Pyroptosis in VSMCs compromises fibrous cap integrity and increases the risk of rupture, ultimately triggering cardiovascular events. This review systematically examines the roles and regulatory features of pyroptosis in these three cell types, aiming to clarify their stage-specific contributions, underlying mechanisms, and potential as therapeutic targets in AS.

### 4.1 The role of ECs pyroptosis in AS

ECs located in the intimal layer of arteries, form a critical barrier of the vascular wall and play essential roles in maintaining vascular homeostasis, regulating permeability, suppressing inflammatory responses, and preventing thrombosis. During the initiation and progression of AS, EC injury and dysfunction serve as key early events (van der Vorst et al., 2015). Activation of EC pyroptosis leads to the release of large quantities of pro-inflammatory cytokines and upregulation of chemokines and adhesion molecules such as intercellular adhesion molecule-1 (ICAM-1) and vascular cell adhesion molecule-1 (VCAM-1), thereby exacerbating vascular inflammation, enhancing leukocyte adhesion, and promoting foam cell formation (Yin et al., 2024). Pyroptosis in ECs facilitates the recruitment of monocytes, macrophages, and T lymphocytes, which subsequently accumulate in the arterial wall and release inflammatory mediators. This process sustains and amplifies chronic inflammation, ultimately contributing to the formation and progression of atherosclerotic plaques (Ju et al., 2022).

Multiple pathological factors can induce EC pyroptosis via distinct signaling pathways and promote AS development. Under inflammatory conditions, elevated levels of IL-1β and IL-18, both products and amplifiers of pyroptosis, can further enhance pyroptotic activity through activation of the nuclear factor kappa B (NF-κB) signaling pathway (Zheng et al., 2020). Oxidative stress (OS) is another critical inducer of EC pyroptosis. Reactive ROS can upregulate the expression of N6-methyladenosine (m6A) methyltransferase METTL3, thereby enhancing the activation of the NLRP3 inflammasome and accelerating the pyroptotic process in ECs (Yang et al., 2023).

Metabolic disorders, particularly hyperlipidemia and hyperglycemia, significantly disrupt EC homeostasis and elevate the risk of pyroptosis. Hyperlipidemia induces EC pyroptosis through the caspase-1/sirtuin-1 activating protein-1 signaling pathway (Yin et al., 2015). Cholesterol crystals markedly activate the NLRP3 inflammasome, intensify pyroptotic responses, and suppress endothelial nitric oxide synthase (eNOS) expression, impairing vasodilation. Hyperglycemia enhances pyroptotic signaling by upregulating E74-like factor 3 (ELF3) and downregulating histone methyltransferase SET8 expression (Wang et al., 2020). Trimethylamine-N-oxide (TMAO), a metabolite of phosphatidylcholine produced by gut microbiota, has been identified as an independent risk factor for coronary heart disease (Zhou W. et al., 2020). TMAO induces EC pyroptosis via the ROS/TXNIP/NLRP3 pathway and promotes the overexpression of succinate dehydrogenase subunit B (SDHB), leading to mitochondrial dysfunction, endothelial impairment, and accelerated atherosclerotic plaque formation (Wu et al., 2020).

Unhealthy lifestyle factors, such as smoking and poor dietary habits, may promote ECs pyroptosis by enhancing inflammasome activity. In ApoE^−/−^ mice exposed to nicotine, more severe atherosclerotic lesions are observed, accompanied by elevated serum levels of IL-1β and IL-18. Mechanistically, nicotine promotes EC pyroptosis and dysfunction via upregulation of ROS and subsequent activation of the thioredoxin-interacting protein (TXNIP)/NLRP3 signaling pathway (Wu et al., 2018).

Mechanical factors, including disturbed hemodynamics and low shear stress (LSS), also contribute to EC pyroptosis. Oscillatory blood flow can trigger pyroptosis through the ROS/NLRP3/caspase-1 pathway and accelerate the development of AS (Chien et al., 2021). LSS promotes pyroptosis via the translocation methylcytosine dioxygenase 2 (TET2)/SDHB/ROS signaling axis and influences vascular remodeling through the NLRP3/STAT3 pathway, thereby increasing the risk of plaque rupture (Chen et al., 2021). Notably, microRNA-181b-5p exerts a protective role by inhibiting the NLRP3/STAT3 axis and suppressing pyroptosis, whereas LSS downregulates miR-181b-5p expression, potentially impairing this protective mechanism during AS progression (Xu X. et al., 2021).

Environmental pollutants are also capable of inducing EC pyroptosis through diverse mechanisms, thereby accelerating AS progression. Heavy metals such as cadmium and hexavalent chromium interfere with EC function by modulating ROS production, autophagy, and inflammasome activation. Cadmium exposure significantly elevates intracellular ROS levels in human umbilical vein endothelial cells (HUVECs) and exacerbates pyroptosis via upregulation of proprotein convertase subtilisin/kexin type 9 (PCSK9) (Zeng et al., 2020). Fibroblast growth factor 21 (FGF21) has been shown to inhibit pyroptosis and attenuate AS progression by modulating the ubiquinol-cytochrome c reductase core protein I (UQCRC1)/ROS pathway. In addition, dietary pollutants such as acrolein can induce EC pyroptosis by stimulating ROS production, suppressing autophagy, and activating the NLRP3 inflammasome (Srivastava et al., 2011). In HUVECs, acrolein exposure increases ROS levels and simultaneously triggers both autophagy and pyroptosis, leading to impaired cell migration (Tang et al., 2017). In EA. hy926 cells, acrolein exerts a dual regulatory effect by modulating ROS levels to activate NLRP3 inflammasomes while enhancing autophagy (Jiang et al., 2018). Persistent organic pollutants, particularly polychlorinated biphenyls (PCBs), are closely associated with increased AS risk. Among them, PCB-118 induces ROS production via aryl hydrocarbon receptor (AhR) activation, subsequently promoting NLRP3 inflammasome activation and exacerbating EC pyroptosis (Tang et al., 2017).

### 4.2 The role of macrophages pyroptosis in AS

Macrophages are among the earliest immune cells to infiltrate the vascular wall and mediate inflammatory responses during the initiation and progression of AS (Neels et al., 2023). Following endothelial dysfunction, circulating monocytes migrate into the subendothelial space and differentiate into macrophages. In early atherosclerotic lesions, macrophages engulf ox-LDL and transform into foam cells, thereby amplifying the release of pro-inflammatory cytokines. The formation of foam cells is considered a hallmark event in the early stages of AS development (Dickhout et al., 2008). As the disease progresses, macrophages exhibit distinct polarization states, including pro-inflammatory (M1) and anti-inflammatory (M2) phenotypes. In advanced atherosclerotic plaques, inflammatory responses mediated by macrophages are markedly intensified. IL-1β and IL-18, released during pyroptosis, can further activate NF-κB signaling pathway, thereby promoting M1 macrophage polarization and the release of additional pro-inflammatory mediators. This leads to a cascading amplification of inflammation within the plaque. In addition to releasing cytoplasmic contents, pyroptotic macrophages secrete extracellular matrix (ECM)-degrading enzymes and matrix metalloproteinases (MMPs), which destabilize the fibrous cap by promoting collagen degradation. This weakens plaque integrity and increases the risk of plaque rupture (Huang et al., 2023). Moreover, pyroptosis is characterized by dramatic plasma membrane rupture and inflammatory cytokine release, which not only induces foam cell death but also triggers secondary pyroptosis in neighboring cells, accelerating the formation of the necrotic core (Shi et al., 2017). Expansion of the necrotic core further compromises fibrous cap stability, rendering atherosclerotic plaques more prone to rupture and ultimately leading to serious cardiovascular events such as acute coronary syndrome (ACS) (Bos et al., 2021).

During the progression of AS, endogenous stimuli such as cholesterol crystals, ox-LDL, and saturated fatty acids (e.g., palmitic acid and stearic acid) serve as potent inducers of macrophage pyroptosis (L'Homme et al., 2013). Cholesterol crystals activate the NLRP3 inflammasome by inducing lysosomal rupture and the subsequent release of cathepsin B (CTSB), which in turn triggers caspase-1–dependent pyroptosis. In macrophages deficient in NLRP3 or its adaptor protein ASC, cholesterol crystals fail to IL-1β release. Moreover, NLRP3-knockout mice exhibit significantly smaller atherosclerotic lesions and lower serum levels of IL-1β and IL-18, highlighting the pivotal role of cholesterol crystal-induced pyroptosis in AS progression (Duewell et al., 2010). Ox-LDL promotes macrophage pyroptosis via the cluster of differentiation 36 (CD36) receptor by inhibiting the nuclear translocation of nuclear factor erythroid 2–related factor 2 (Nrf2), thereby suppressing the expression of heme oxygenase-1 (HO-1) and NADPH quinone oxidoreductase-1 (NQO-1). This leads to increased ROS production, activation of the NLRP3 inflammasome, and subsequent caspase-1–mediated pyroptosis (Ji et al., 2021). Palmitic acid can impair mitophagy through NLRP3 inflammasome activation, resulting in mitochondrial ROS accumulation and accelerated pyroptotic death of macrophages (L'Homme et al., 2013). Metabolic dysregulation, particularly enhanced glycolysis, also contributes to macrophage pyroptosis. Upon prolonged stimulation with ox-LDL, monocyte-derived macrophages display increased glycolytic flux and activation of the absent in melanoma 2 (AIM2) inflammasome, leading to expansion of the necrotic core within atherosclerotic plaques (Du et al., 2022). NIP3-like protein X (NIX), a mitochondrial outer membrane protein, is involved in mitophagy and plays a regulatory role in pyroptosis. NIX-mediated mitophagy reduces ROS production and preserves mitochondrial integrity, thereby suppressing the NLRP3/caspase-1/IL-1β pathway. Studies have shown that silencing NIX significantly enhances ox-LDL–induced macrophage pyroptosis, suggesting that ox-LDL may promote pyroptosis by inhibiting NIX-dependent mitophagy (Peng et al., 2020). Pro-inflammatory lipid mediators such as lysophosphatidylcholine (LPC) and oxidized phosphatidylcholine (oxPAPC) are also implicated in macrophage pyroptosis. LPC activates the NLRP3 inflammasome by inducing lysosomal damage and intracellular potassium efflux, thereby triggering pyroptotic cell death (Liang et al., 2024). OxPAPC enhances mitochondrial ROS generation and IL-1β secretion, further aggravating local inflammation (Kadl et al., 2011). NINJ1 is a newly identified executor protein of pyroptosis and a substrate of MMP9. Its expression is significantly elevated in arterial macrophages of ApoE^−/−^ mice. Soluble NINJ is detectable in the serum of AS patients and has been proposed to modulate monocyte recruitment and regulate the progression of AS (Jeon et al., 2020).

Exogenous factors such as bacterial infections, environmental pollutants (e.g., PM2.5), and nicotine have also been implicated in the induction of macrophage pyroptosis. Certain bacterial infections are recognized as important external risk factors for AS. For example, *Chlamydia pneumoniae* can induce macrophage pyroptosis by activating the Toll-like receptor 2 (TLR2) signaling pathway, thereby promoting foam cell formation (Sessa et al., 2009). Cell wall extracts of *Lactobacillus casei* have been shown to activate caspase-1 and CD11c^+^ macrophages, triggering the release of IL-1β and IL-18 and exacerbating local inflammatory responses (Lee et al., 2012). Infection with *Porphyromonas gingivalis* (Pg), a periodontal pathogen, promotes activation of the NLRP3 inflammasome in macrophages via the CD36/TLR2 pathway, further enhancing IL-1β and IL-18 secretion and accelerating the progression of AS (Brown et al., 2015). Nicotine, the principal bioactive component of tobacco, can increase macrophage pyroptosis by promoting ROS production and activating the TXNIP/NLRP3/caspase-1 signaling cascade. In ApoE^−/−^ mice, nicotine exposure results in more severe atherosclerotic lesions and increased lipid accumulation within plaques (Xu S. et al., 2021). Interestingly, extracellular pH and environmental conditions may also influence AS progression. Macrophages isolated from mice exposed to PM2.5 display increased NLRP3 expression and caspase-1 activity, suggesting that PM2.5 promotes macrophage pyroptosis and contributes to AS development (Wang et al., 2021). These findings underscore the importance of considering environmental exposures as potential modulators of pyroptosis in the prevention and treatment of AS.

### 4.3 The role of VSMCs pyroptosis in AS

VSMCs predominantly located in the tunica media, exhibit considerable plasticity. Under physiological conditions, VSMCs maintain a contractile phenotype, contributing to the regulation of vascular tone and elasticity. However, in response to pathological stimuli such as inflammatory cytokines or lipid deposition, VSMCs undergo phenotypic switching—from a contractile phenotype to a synthetic phenotype—and may further transdifferentiate into macrophage-like or osteoblast-like cells (Ju et al., 2022). Phenotypically modulated VSMCs migrate to the intimal layer and, together with the ECM they secrete, including collagen and elastin, form the major structural component of the fibrous cap in atherosclerotic plaques (Burger et al., 2021). Loss of VSMCs and degradation of ECM weaken the fibrous cap, thereby compromising plaque composition and stability, and significantly increasing the risk of rupture (Bennett et al., 2016).

Studies have demonstrated that VSMC pyroptosis is mediated by multiple inflammasomes, with NLRP3 and AIM2 playing central roles in this process (Xu et al., 2022). In human carotid artery plaque specimens, the NLRP3 inflammasome has been shown to directly promote the phenotypic transformation of VSMCs into macrophage-like cells, thus contributing to plaque destabilization. High concentrations of ox-LDL can induce VSMC pyroptosis by activating the NF-κB signaling pathway and upregulating AIM2 expression (Akishima et al., 2005). Animal studies have further confirmed that AIM2 overexpression in ApoE^−/−^ mice significantly enhances VSMC pyroptosis and accelerates AS progression (Fidler et al., 2021).

The ECM synthesized by VSMCs plays a protective role in AS by shielding the necrotic core of plaques, thereby reducing the likelihood of plaque rupture. However, pyroptosis results not only in reduced VSMC numbers but also in impaired ECM production, leading to fibrous cap thinning and increased vulnerability to rupture (Harman and Jørgensen, 2019). Notably, in human atherosclerotic plaques, elevated expression of pyroptosis-related proteins—including NLRP3, ASC, and GSDMD—is closely associated with VSMC loss. Treatment of ApoE^−/−^ mice with the caspase-1 inhibitor VX-765 significantly reduces VSMC pyroptosis and enhances fibrous cap stability, underscoring the critical role of VSMC pyroptosis in plaque destabilization (Li et al., 2020). Enhanced VSMC pyroptosis also leads to increased secretion of ECM-degrading enzymes, such as MMPs, which further compromise the mechanical strength of the fibrous cap and exacerbate plaque instability (Li et al., 2024). In addition, adiponectin (APN), an adipose tissue–specific protein with anti-inflammatory and anti-atherosclerotic properties, exerts a protective effect by upregulating miR-133a and inhibiting NLRP3 inflammasome activation, thereby attenuating VSMC pyroptosis (Duan et al., 2020). Furthermore, lipopolysaccharide derived from Pg-LPS has been shown to promote VSMC proliferation, activate caspase-1, and induce IL-1β and IL-18 secretion, ultimately accelerating VSMC pyroptosis and compromising plaque stability (Liu J. et al., 2021).

## 5 Mechanisms of pyroptosis regulation by natural products in AS

As a rich source of natural compounds, TCM contains a variety of bioactive phytochemicals, such as polyphenols and saponins, which have been extensively studied and shown to exert multi-targeted and systemic effects against AS by modulating pyroptosis-related signaling pathways. We posit that a pyroptosis-targeted intervention strategy based on TCM-derived active compounds offers a distinct advantage in regulating immune-inflammatory responses, oxidative stress, and programmed cell death, reflecting a superior capacity for network-level modulation. This approach holds significant potential for clinical translation. Therefore, this review systematically summarizes recent advances in the use of plant-derived compounds and TCM formulations for the treatment of AS via regulation of pyroptosis, with a particular focus on their molecular mechanisms, cellular targets, and therapeutic advantages. The aim is to provide a theoretical basis and research perspective for developing novel anti-AS strategies centered on pyroptosis modulation.

### 5.1 Medicinal plant-derived compounds

**FIGURE 2:**
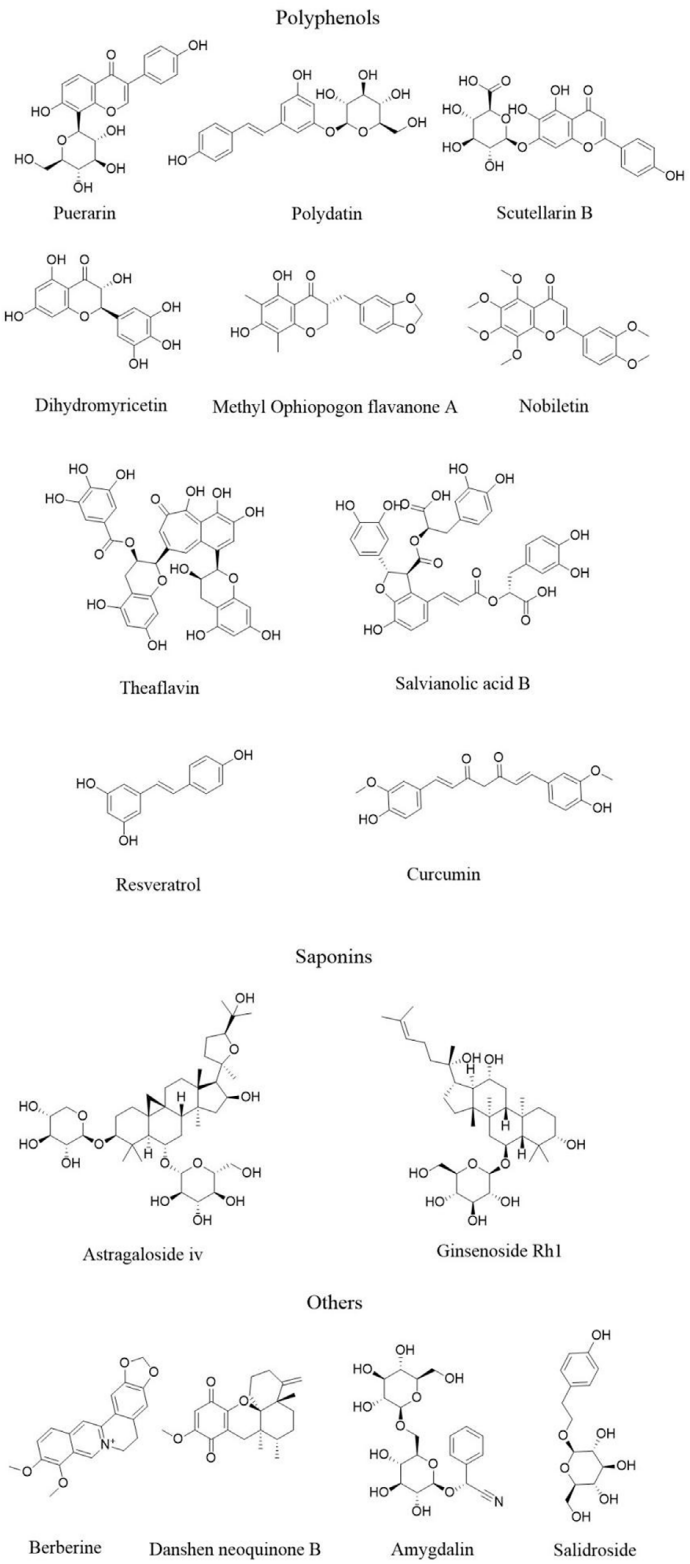
The structure of active ingredients of natural products.

Plant-derived natural products, including polyphenols, flavonoids, and saponins, have been confirmed by numerous studies to exert anti-AS effects by modulating pyroptosis-related signaling pathways. These natural products demonstrate substantial potential in regulating AS through their ability to modulate immune-inflammatory responses, oxidative stress, and cell death pathways, offering important prospects for clinical translation. Therefore, this section systematically reviews recent advances in the use of natural products to target pyroptosis pathways for the prevention and treatment of AS. The mechanisms of these natural products are summarized in Table 1, and their chemical structures are presented in Figure 2.

**TABLE 1:** Natural products targeting pyroptosis in atherosclerosis.

Type of extract		Herbal extracts	Plant	Mechanism and effect	Model		References
					*In vivo*	*In vitro*	
Polyphenols	Isoflavones	Puerarin	*Pueraria lobata* (Willd.) Ohwi [Fabaceae]	Downregulated the expression of NLRP3, caspase-1, cleaved-caspase-1 and TNF -α, and inhibited the nlrp3/caspase-1 signaling pathway.	-	THP-1 cells	Zhang et al. (2021)
		Polydatin	*Polygonum cuspidatum* (Siebold & Zucc.) [Polygonaceae], *Polygonum multiflorum* Thunb. [Polygonaceae]	It inhibited the co localized expression of tunel/caspase 1 and f4/80 NLRP3, inhibited the recruitment of ASC and caspase-1 and gsdmd-n expression, and decreased the phosphorylation of NLRP3 and mTOR and the expression level of p62.	ApoE−/−mice	-	Zhang et al. (2023)
				Downregulation of tnf-αTREM-1, ROS, LDH, Caspase-1, NLRP3, ASC, GSDMD. Regulation of trem-1/nlrp3/caspase-1 signaling pathway.	-	HUVECs cells	Kong (2024)
	Flavonoids	Scutellarin	*Erigeron breviscapus* (Vant.) Hand.-Mazz. [Asteraceae]	Downregulate the levels of NLRP3, ASC, pro-caspase-1, caspase-1, gsdmd NT, and inhibit the nlrp3/caspase-1 signaling pathway.	-	HUVECs cells	Yang and Shen (2022)
		Dihydromyricetin	*Ampelopsis grossedentata* (Wall.) W.T. Aiton [Vitaceae], *Myrica rubra* Siebold & Zucc. [Myricaceae], *Citrus aurantium* L. [Rutaceae]	Downregulate the expression of NLRP3, casp1p20, IL-1β, ICAM-1, upregulate the expression of Nrf2, HO-1, NQO1, regulate ROS levels through Nrf2 signaling pathway, and then inhibit vascular ECS pyroptosis.	-	HUVECs cells	Hu et al. (2018)
	High isoflavones	Methyl Ophiopogon flavanone A	*Ophiopogon japonicus* (Thunb.) Ker Gawl. [Asparagaceae]	It can inhibit the activities of SOD and cat, reduce ROS production, downregulate the expression of NLRP3, caspase 1, IL-1β, n-gsdmd, and inhibit LPS/ATP induced pyroptosis through ros/nlrp3 pathway.	-	macrophages	Zeng et al. (2023)
	Polymethoxy flavonoids	Nobiletin	*Citrus reticulata* Blanco [Rutaceae]	Upregulate the expression of LC3Ⅱ, belin1, PINK1 and Parkin, downregulate the expression of gsdmd, NLRP3, ASC, caspase-1 and p62, activate PINK1/parkin mediated mitophagy to maintain mitochondrial homeostasis, and inhibit the activation of NLRP3 inflammasome.	ApoE−/−mice	macrophages	Deng (2021)
	Biflavonoids	Theaflavin	*Camellia sinensis* L. [Theaceae]	Downregulated the expression of gsdmd, NLRP3 and caspase-1.	ApoE−/−mice	macrophages	Xia (2022)
	Anthraquinone terpenoids	Resveratrol	*Vitis vinifera* L. [Vitaceae], *Polygonum cuspidatum* (Siebold & Zucc.) [Polygonaceae]	Downregulate the expression of BMP2, Runx2, NLRP3, caspase-1, gsdmd, and inhibit the nlrp3/caspase-1 signaling pathway.	SD rats	CRL-1999 cells	Li et al. (2022b)
	Diarylheptanoids	Curcumin	*Curcuma longa* L. [Zingiberaceae], *Curcuma zedoaria* (Christm.) Rosc. [Zingiberaceae], *Curcuma aromatica* Salisb	Upregulated the expression of uqcrc1 and tet2, downregulated the expression of uqcrc1, caspase-1 and NLRP3, restoring αvβ3 and reducing endothelin-1 expression	-	HUVECs cells	Yuan et al. (2022)
	Phenolic acids	Salvianolic acid B	*Sal*via *miltiorrhiza* Bunge [Lamiaceae]	Downregulated the expression of ROS, caspase-1 and NLRP3, and regulated the ampk/foxo4/klf2 and syndecan-4/rac1/atf2 pathways.	-	Endothelial progenitor cells	Tang et al. (2022)
Saponins	Tetracyclic triterpenoid saponins	Astragaloside IV	*Astragalus membranaceus* (Fisch.) Bunge [Fabaceae]	It upregulated Bcl-2 and Bax expression, downregulated NLRP3, caspase-1 and ASC expression, and regulated ROS/NLRP3 mediated apoptosis of ECs.	-	HUVECs	Su et al. (2022b)
				It downregulated map3k8, p-nf-κB p65, p-JNK, NLRP3, ASC, aim2, caspase-1, gsdmd, upregulated tgf-β, Arg-1, STAT6, and regulated map3k8 mediated pyroptosis in macrophages.	ApoE−/−mice	-	He et al. (2023)
	Triterpenoid saponins	Ginsenoside Rh1	*Panax ginseng* C.A. Meyer [Araliaceae], *Panax quinquefolius* L. [Araliaceae], *Panax notoginseng* (Burk.) F.H. Chen [Araliaceae], *Eleutherococcus senticosus* (Rupr. & Maxim.) Maxim. [Araliaceae]	Downregulated the levels of MDA, IL-1βand tnf-α, restored the activities of SOD and cat, and regulated the expression of HSP70/NF -κB p65.	ApoE−/−mice	HUVECs cells	Song (2024)
Others	Alkaloid	Berberine	*Coptis chinensis* (Franch.) [Ranunculaceae]	Downregulated mtROS, NLRP3, IL-1 β, LDH and NLRP3, and reduce the activation level of caspase-1 and its mediated pyroptosis rate.	-	HUVECs cells	Su et al. (2022a)
	Quinone compounds	Danshen neoquinone B	*Sal*via *miltiorrhiza* Bunge [Lamiaceae]	Downregulated the expression of nf-κB1, NLRP3, gsdmd, IL-1β through nf-κb/nlrp3 signaling pathway.	-	HUVECs cells	Li et al. (2023)
	Cyanogenic glycosides	Amygdalin	*Prunus armeniaca* L. [Rosaceae]	Downregulate the expression of Caspase-1, gsdmd, Gal-3, jmjd3, upregulate the level of H3K27me3, regulate jmjd3 mediated demethylation of Gal-3 on as induced pyroptosis.	ApoE−/−mice	HCAECs cells	Liu et al. (2023)
	Phenylethanol glycosides	Salidroside	*Rhodiola rosea*L. [Crassulaceae]	Downregulation of HIF-1α, Caspase1, Gsdmd-N, NRLP3, IL-18 and IL-1β, Regulation of macrophages function by binding to HIF-1α.	ApoE−/−mice	-	Guo et al. (2025)

#### 5.1.1 Polyphenols

Polyphenolic compounds are widely distributed in plants and exhibit a broad spectrum of chemical structures, typically consisting of one or more hydroxyl groups attached to aromatic rings. Based on their structural characteristics, polyphenols can be classified into several major categories, including flavonoids, phenolic acids, stilbenes, and lignans (Yahfoufi et al., 2018). Polyphenols possess potent anti-inflammatory, antioxidant, and immunomodulatory properties, and have demonstrated beneficial roles in the prevention and attenuation of chronic cardiovascular diseases, including AS (Santhakumar et al., 2018; Ziółkiewicz et al., 2023).

Flavonoids typically possess a basic C6-C3-C6 skeleton and are further subclassified into flavones, flavonols, flavanones, flavanols, isoflavones, and anthocyanins based on their structural variations (Liu W. et al., 2021). Puerarin, an isoflavone extracted from the root of the leguminous plant *Pueraria lobata* (Willd.) Ohwi [Fabaceae], has been shown to inhibit ox-LDL-induced lipid accumulation and foam cell formation in macrophages. It dose-dependently downregulates pyroptosis-related proteins such as NLRP3 and caspase-1, as well as proinflammatory cytokines, thereby contributing to the stabilization of vulnerable atherosclerotic plaques (Zhang et al., 2021). Polydatin, a natural stilbenoid glycoside primarily found in *Polygonum cuspidatum* (Siebold & Zucc.) [Polygonaceae] and *Polygonum multiflorum Thunb. [Polygonaceae]*, improves lipid profiles and reduces inflammatory cytokine expression in ApoE^−/−^ atherosclerotic mice. It inhibits macrophage pyroptosis by downregulating TUNEL/caspase-1 and F4/80/NLRP3 co-expression, decreasing phosphorylated NLRP3 and mTOR, and suppressing p62 expression (Zhang et al., 2023). Polydatin also inhibits LPS-induced proliferation, migration, and adhesion of HUVECs, and suppresses pyroptosis by modulating the TREM-1/NLRP3/caspase-1 signaling pathway (Kong, 2024). Scutellarin (Scu), a flavonoid abundantly present in *Erigeron breviscapus (Vant.) Hand.-Mazz. [Asteraceae]*, inhibits activation of the NLRP3 inflammasome in a dose-dependent manner by downregulating the expression of NLRP3, ASC, pro-caspase-1, caspase-1, and GSDMD-N, thereby reducing IL-1β and IL-18 release. It protects HUVECs from LPS + ATP–induced inflammation and pyroptotic injury (Yang and Shen, 2022). Methylophiopogonanone A (MO-A), an isoflavone derived from *Ophiopogon japonicus (Thunb.) Ker Gawl. [Asparagaceae]*, attenuates LPS/ATP-induced macrophage pyroptosis by reducing ROS production, enhancing SOD and CAT activity, and downregulating NLRP3, caspase-1, IL-1β, and GSDMD expression. These findings suggest that MO-A may suppress macrophage pyroptosis and AS-related inflammation via modulation of the ROS/NLRP3 pathway (Zeng et al., 2023). Dihydromyricetin (DHM), is a natural flavonoid compound widely found in the medicinal and edible plants *Ampelopsis grossedentata* (Wall.) W.T. Aiton [Vitaceae], *Myrica rubra* Siebold & Zucc. [Myricaceae], and *Citrus aurantium* L. [Rutaceae]. DHM downregulates the expression of NLRP3, caspase-1 p20, IL-1β, and ICAM-1, upregulating Nrf2, HO-1, and NQO1, while significantly improves endothelial cell viability and membrane integrity. These results indicate that DHM may inhibit endothelial pyroptosis through Nrf2-mediated regulation of intracellular ROS and mitochondrial ROS (mtROS) levels (Hu et al., 2018). Nobiletin (NOB), polymethoxylated flavonoid abundant in the plant *Citrus reticulata* Blanco [Rutaceae], reduces foam cell lipid accumulation and ROS production in ApoE^−/−^ mice. It activates PINK1/Parkin–mediated mitophagy to restore mitochondrial homeostasis and inhibits NLRP3 inflammasome activation, thereby exerting anti-pyroptotic and anti-atherosclerotic effects (Deng, 2021). Theaflavins (TFs), a class of dimeric polyphenols predominantly present in *Camellia sinensis* L. [Theaceae], dose-dependently inhibit ox-LDL–induced expression of pyroptosis-related proteins including GSDMD, NLRP3, caspase-1, IL-1β, and IL-18 in macrophages. TFs also significantly reduce lipid deposition in the arterial intima of ApoE^−/−^ mice (Xia, 2022).

Resveratrol (Res), a natural stilbene compound, can be extracted from *Vitis vinifera* L. [Vitaceae] and *Polygonum cuspidatum* (Siebold & Zucc.) [Polygonaceae]. It exhibits anti-inflammatory, antioxidant, anti-fibrotic, and cardioprotective properties (Voloshyna et al., 2012). Resveratrol enhances atherosclerotic plaque stability and delays AS progression by modulating the NLRP3/caspase-1 signaling pathway. It downregulates the expression of Runx2, BMP2, and other calcification-related markers, thereby inhibiting the osteogenic transdifferentiation of VSMCs, suppressing VSMC pyroptosis and associated inflammatory responses (Li S. et al., 2022).

Curcumin is a diarylheptanoid polyphenolic compound primarily found in the plants *Curcuma longa* L. [Zingiberaceae], *Curcuma zedoaria* (Christm.) Rosc. [Zingiberaceae], and *Curcuma aromatica* Salisb. [Zingiberaceae]. It has well-documented antioxidant, anti-inflammatory, and anti-AS effects (Singh et al., 2021). Studies have shown that curcumin upregulates the expression of UQCRC1 and 10–11 TET2, while downregulating pyroptosis-related molecules including caspase-1, NLRP3, and IL-1β, endothelin-1 (Yuan et al., 2022). It partially reverses TMAO-induced ROS generation and attenuates HUVEC pyroptosis and mitochondrial dysfunction (Zhao, 2020).

Salvianolic acid B (SalB) is a phenolic acid and one of the major bioactive components of *the plant Sal*via *miltiorrhiza Bunge [Lamiaceae],* which has been widely used in the treatment of cardiovascular diseases. SalB suppresses ROS accumulation and modulates the AMPK/FoxO4/KLF2 and Syndecan-4/Rac1/ATF2 signaling pathways. Through these mechanisms, it mitigates endoplasmic reticulum stress and inhibits TXNIP/NLRP3 inflammasome-mediated pyroptosis in endothelial cells, thereby improving endothelial injury and dysfunction and slowing the progression of AS (Tang et al., 2022).

#### 5.1.2 Saponins

Saponins are a class of naturally occurring glycosides widely found in plants. Based on their chemical structures, they are mainly categorized into triterpenoid saponins and steroidal saponins. These compounds exhibit a range of biological activities, including anti-inflammatory, antiviral, immunomodulatory, cardioprotective, and anticancer effects (Luo et al., 2022). Astragaloside IV (AS-IV) is a naturally occurring saponin compound extracted from the plant *Astragalus membranaceus* (Fisch.) Bunge [Fabaceae]. AS-IV significantly downregulates the expression of key pyroptosis-related proteins such as NLRP3, caspase-1, and ASC, while increasing the B-cell lymphoma-2 (BCL-2) to BCL2-associated X protein (BAX) ratio and reducing intracellular ROS levels. Through modulation of multiple signaling pathways, AS-IV suppresses inflammation and inhibits LPS-induced pyroptosis in HUVECs (Su Y. et al., 2022). Additionally, AS-IV exerts anti-atherosclerotic effects by regulating macrophage polarization. It downregulates the expression of MAP3K8, phosphorylated NF-κB p65, phosphorylated JNK, NLRP3, ASC, AIM2, caspase-1, and GSDMD, while upregulating transforming growth factor-β (TGF-β), arginase-1 (Arg-1), and STAT6, thereby promoting M2-type macrophage polarization and reducing proinflammatory responses mediated by M1-type macrophages (He et al., 2023). Ginsenosides are primarily extracted from the medicinal plants *Panax ginseng* C.A. Meyer [Araliaceae], *Panax quinquefolius* L. [Araliaceae], *Panax notoginseng* (Burk.) F.H. Chen [Araliaceae], and *Eleutherococcus senticosus* (Rupr. & Maxim.) Maxim. [Araliaceae]. Ginsenosides also demonstrate cardiovascular protective potential. Ginsenoside Rh1 ameliorates vascular endothelial injury induced by polystyrene nanoplastics (Nps) in mice, significantly reducing intimal thickening and plaque formation in the aortic root, along with decreased collagen fiber deposition. Mechanistically, Rh1 modulates the expression of heat shock protein 70 (HSP70) and NF-κB p65, inhibits excessive activation of the endoplasmic reticulum stress-related PERK pathway, and suppresses endothelial cell pyroptosis, thereby exerting protective effects against AS (Song Y., 2024).

#### 5.1.3 Other compounds

Berberine (BBR) is a benzylisoquinoline quaternary ammonium alkaloid primarily derived from *Coptis chinensis* (Franch.) [Ranunculaceae]. BBR could reduce the generation of mtROS induced by H_2_O_2_ in macrophages, downregulate the expression levels of NLRP3, IL-1 β and NLRP3, reduce the release of LDH and IL-1 β in cell supernatants, and reduce the activation level of caspase-1 and its mediated pyroptosis rate. This suggests that berberine may inhibits H_2_O_2_-induced pyroptosis of macrophages through mtROS NLRP3 pathway (Su R. et al., 2022). Tanshinone B, a diterpene quinone compound extracted from *Sal*via *miltiorrhiza* Bunge [Lamiaceae], exerts anti-atherosclerotic effects by downregulating the protein and mRNA expression levels of NF-κB1, NLRP3, GSDMD, and IL-1β. It suppresses HUVEC pyroptosis by inhibiting NLRP3 inflammasome activation through the NF-κB/NLRP3 signaling axis (Li et al., 2023). Amygdalin (AMY), a cyanogenic glycoside found in the seeds of *Prunus armeniaca* L. [Rosaceae], mitigates aortic lipid deposition and plaque formation in high-fat diet (HFD)-induced ApoE^−/−^ mice. Mechanistically, AMY downregulates the expression of caspase-1, GSDMD, galectin-3 (Gal-3), and JMJD3, while upregulating trimethylation at histone H3 lysine 27 (H3K27me3). It modulates JMJD3-mediated demethylation of Gal-3, thereby suppressing pyroptosis associated with AS (Liu et al., 2023). Salidroside (SAL), a phenylethanoid glycoside extracted from *Rhodiola rosea*L. [Crassulaceae], has been shown to reduce lipid accumulation in macrophages and improve atherosclerotic plaque formation by inhibiting the pyroptosis pathway mediated by hypoxia-inducible factor 1 alpha (Hif-1α). In a study using ApoE^−/−^ mice and macrophage-derived foam cell models, the direct binding between SAL and Hif-1α was validated. The results demonstrated that SAL not only suppressed Caspase-1-dependent pyroptosis but also reduced plaque area *in vivo*, improved lipid metabolism, and downregulated the expression of Hif-1α, NLRP3, Caspase-1, GSDMD, and related inflammatory cytokines (Guo et al., 2025).

Current research on the regulation of pyroptosis by natural products in AS has yielded substantial findings, yet notable limitations remain. First, the choice of experimental models is relatively narrow, with most studies relying on ApoE^−/−^ or high-fat diet–induced mouse models, and lacking validation in more diverse atherosclerosis models, such as animals with knockdown of NLRP3 or pyroptosis-related inflammatory mediators. Some compounds (puerarin, scutellarin, curcumin, berberine) have been evaluated only at the cellular level, without *in vivo* confirmation of consistency. Second, mechanistic investigations often focus on measuring the expression of NLRP3, caspase-1, GSDMD, and inflammatory cytokines, but rarely establish a closed causal loop using gene knockout or conditional knockout models. Detection methods are largely limited to Western blotting or immunofluorescence, with few studies employing functional assays for membrane pore formation (LDH release, membrane permeability imaging) to confirm pyroptosis. Although polyphenols (puerarin, resveratrol, theaflavins, salvianolic acid B), saponins (astragaloside IV, ginsenosides), and other compounds (berberine, tanshinone B, salidroside) demonstrate multi-target benefits—such as inhibiting NLRP3 inflammasome activation, improving mitochondrial homeostasis, and modulating macrophage polarization—most studies have not clearly distinguished between their anti-inflammatory or antioxidant effects and their direct inhibition of pyroptosis. The precise relationship between these natural products and canonical pyroptotic pathways therefore warrants further elucidation.

### 5.2 Traditional Chinese Medicine

In the preceding section, we focused on the research progress of isolated natural products in regulating pyroptosis and preventing AS. However, in clinical practice, the application of natural products is not limited to single compounds. TCM formulas also constitute an important component of natural products and have a long and rich history in the prevention and treatment of cardiovascular diseases. Characterized by their multi-component, multi-target, and synergistic therapeutic properties, TCM formulas are particularly well suited to modulating the complex pathological processes of pyroptosis, which involve multiple signaling pathways and diverse cell types in AS. In recent years, studies have begun to reveal the regulatory effects of specific TCM formulas on key molecular events in pyroptosis, providing new strategies for the prevention and treatment of atherosclerotic lesions. Table 2 summarizes the currently reported TCM formulas, compositions, and mechanisms of action targeting pyroptosis in the treatment of AS.

**TABLE 2:** TCM targeting pyroptosis in atherosclerosis.

Traditional Chinese medicine preparations	Component	Range tested	Model		Control		Treatmenttime	Mechanism and effect	References
					Control group	Experimental group			
Gegen Qinlian Decoction	*Pueraria lobata* (Willd.) Ohwi [Fabaceae], *Scutellaria baicalensis* Georgi [Lamiaceae], *Coptis chinensis* (Franch.) [Ranunculaceae], and *Glycyrrhiza uralensis* Fisch. [Fabaceae].	0.104/g/mL/d, 0.208/g/mL/d, 0.416/g/mL/d	ApoE−/−mice	Establishment of as mouse model by high-fat diet	Blank group, Model group, Western medicine group	Gegen Qinlian Decoction low, middle and high dose groups	8 weeks	Inhibit ASC NLRP3, C-terminalGSDMD, N-terminalGSDMD, pro-Caspase-1 And nf-κbp65 expression, increased the expression of cd206, and inhibited macrophages pyroptosis by regulating the nf-κb/nlrp3/caspase-1 pathway.	Zheng et al. (2022b)
Simiao Yong’an Decoction	*Lonicera japonica* Thunb. [Caprifoliaceae], *Scrophularia ningpoensis* Hemsl. [Scrophulariaceae], *Angelica sinensis* (Oliv.) Diels [Apiaceae], and *Glycyrrhiza uralensis* Fisch. [Fabaceae]	11.61 g/kg/d, 23.14 g/kg/d, 34.9 g/kg/d	ApoE−/−mice	Establishment of as mouse model by high-fat diet	Blank group, Model group, Western medicine group	Simiao Yong’an Decoction low, middle and high dose groups	4 weeks	Inhibit the expression of TLR4, nf-κB, gsdmd, NLRP3, caspase-1, IL-1β, and inhibit pyroptosis by regulating tlr4/nlrp3/caspase-1 signaling pathway.	Yu et al. (2021b)
Jianpi Qutan Huayu Decoction	*Codonopsis pilosula* (Franch.) Nannf. [Campanulaceae], *Atractylodes macrocephala* Koidz. [Asteraceae], *Poria cocos* (Schw.) Wolf [Polyporaceae], *Pinellia ternata* (Thunb.) Breit. [Araceae], *Trichosanthes kirilowii* Maxim. [Cucurbitaceae], *Prunus persica* (L.) Batsch [Rosaceae], *Carthamus tinctorius* L. [Asteraceae], and *Glycyrrhiza uralensis* Fisch. [Fabaceae].	0.195 g/kg/d, 0.586 g/kg/d, 1.758 g/kg/d	ApoE−/−mice	Establishment of as mouse model by high-fat diet	Blank group, Model group, Western medicine group	Jianpi Qutan Huayu Decoction low, middle and high dose groups	12 weeks	Inhibit the expression of NLRP3, caspase-1, gsdmd, pro-caspase-1mrna.	Zhao et al. (2024)
Xinmaikang prescription	*Imperata cylindrica* (L.) Raeusch. [Poaceae], *Curcuma zedoaria* (Christm.) Rosc. [Zingiberaceae], *Trionyx sinensis* (Wiegm.) [Trionychidae], *Citrus aurantium* L. [Rutaceae], *Aristolochia contorta* Bunge [Aristolochiaceae], and *Dendrobium officinale* Kimura & Migo [Orchidaceae].	1.5 g/kg/d, 3.0 g/kg/d	ApoE−/−mice	Establishment of as mouse model by high-fat diet	Model group, Western medicine group	Xinmaikang Prescription low, and high dose groups	12 weeks	Inhibition ICAM-1, IL-18, VCAM-1, MMP-9, NLRP3, MCP-1, TLR4, Caspase-1, IL-1β, GSDMD-N Through regulating the tlr4/nlrp3/caspase-1 pathway, it inhibits macrophages pyroptosis.	He et al. (2025)
Vascular softening pill	*Crataegus pinnatifida* Bunge [Rosaceae], *Citrus reticulata* Blanco [Rutaceae], *Pinellia ternata* (Thunb.) Breit. [Araceae], *Astragalus membranaceus* (Fisch.) Bunge [Fabaceae], *Sal*via *miltiorrhiza* Bunge [Lamiaceae], *Panax notoginseng* (Burk.) F.H. Chen [Araliaceae], and *Raphanus sativus* L. [Brassicaceae].	15.73 g/kg/d, 31.46 g/kg/d	ApoE−/−mice	Establishment of as mouse model by high-fat diet	Blank group, Model group, Western medicine group, AMPK inhibitor group	vascular softening pill low and high dose groups	12 weeks	Increased the expression level of p-ampk, inhibited the expression of NLRP3, cleaved-caspase1, gsdmd-n, IL-1β, IL-18, It alleviates pyroptosis by regulating ampk/nlrp3 pathway.	Qin et al. (2024a)
Zhuyu Pills	*Evodia rutaecarpa* (Juss.) Benth. [Rutaceae] and *Coptis chinensis* (Franch.) [Ranunculaceae].	130.54 mg/kg/d, 261.08 mg/kg/d, 552.16 mg/kg/d	SPF grade male c57bl/6j mice	Establishment of as mouse model by high-fat diet	Blank group, Model group, Western medicine group	Zhuyu Pills low, middle and high dose groups	12weeks	It can reduce the generation of serum TMAO, inhibit the regulation of ros/txnip/nlrp3 signaling pathway, improve the inflammatory response, and reduce the pyroptosis of ECs.	Song et al. (2024)
Qingxin Jieyu Formula	*Astragalus membranaceus* (Fisch.) Bunge [Fabaceae], *Sal*via *miltiorrhiza* Bunge [Lamiaceae], *Ligusticum chuanxiong* Hort. [Apiaceae], *Pogostemon cablin* (Blanco) Benth. [Lamiaceae], and *Coptis chinensis* (Franch.) [Ranunculaceae].	1.05 g/kg/d, 2.1 g/kg/d, 4.2 g/kg/d	ApoE−/−mice	Establishment of as mouse model by high-fat diet	Blank group, Model group, Western medicine group	Qingxin Jieyu Formula low, middle and high dose groups	8 weeks	It can promote the polarization of RAW264.7 macrophages from M1 to M2, and can regulate the Sirt1/PPARγ/NF-κB signaling pathway to inhibit pyroptosis.	Song (2024)
Zuogui Jiangtang Shuxin Formula	*Astragalus membranaceus* (Fisch.) Bunge [Fabaceae], *Rehmannia glutinosa* (Gaertn.) DC. [Orobanchaceae], *Coptis chinensis* (Franch.) [Ranunculaceae], *Sal*via *miltiorrhiza* Bunge [Lamiaceae], *Cornus officinalis* Siebold & Zucc. [Cornaceae], *Crataegus pinnatifida* Bunge [Rosaceae], and *Pueraria lobata* (Willd.) Ohwi [Fabaceae].	25.92 g/kg/d	Mouse j774a.1macrophages	LPS induced pyroptosis of j774a.1macrophages	Blank group, Model group, Western medicine group	5% and 10% Zuogui Jiangtang Shuxin Formula-containing plasma	24 h	Inhibit the expression levels of TLR4, p-nf- κ bp65, NLRP3, gsdmd-n, IL-1 β.	Yang et al. (2022)
Zhilong Huoxue Tongyu Capsule	*Astragalus membranaceus* (Fisch.) Bunge [Fabaceae], *Lumbricus rubellus* Hoffmeister [Lumbricidae], *Tacca palmata* Forst. [Dioscoreaceae], *Cinnamomum cassia* (L.) J.Presl [Lauraceae], and *Hirudo nipponia* (Hirudinidae).	3 μg/mL, 10 μg/mL, 30 μg/mL	U937 cells	U937 monocytes were induced into macrophages by PMA	Blank group, Model group	Zhilong Huoxue Tongyu Capsule low, middle and high dose groups	24 h	By inhibiting the binding of adaptor protein ASC to pro caspase-1, inhibiting Caspase-1 plays an anti pyroptotic role in the cleavage of gsdmd and pro-il-1 β and the protein expression of gsdmd-n and IL-1 β, and inhibits the activation of NLRP3 inflammasome.	Liu et al. (2020a)
Soufeng Qutan Decoction	*Buthus martensii* Karsch [Buthidae], *Scolopendra subspinipes* (Linnaeus) [Scolopendridae], *Lumbricus rubellus* Hoffmeister [Lumbricidae], *Hirudo nipponia* (Hirudinidae), *Citrus reticulata* Blanco [Rutaceae], *Pinellia ternata* (Thunb.) Breit. [Araceae], and *Atractylodes macrocephala* Koidz. [Asteraceae].	6.825 g/kg/d, 13.65 g/kg/d, 27.3 g/kg/d	ApoE−/−mice	Establishment of as mouse model by high-fat diet	Blank group, Model group, Western medicine group	Soufeng Qutan Decoction low, middle and high dose groups	13 weeks	Inhibit the expression levels of NLRP3, caspase-1, ASC, IL-1βand IL-18, reduce plaque area and improve blood lipids.	Zhao et al. (2022)

#### 5.2.1 Gegen Qinlian decoction

Gegen Qinlian Decoction (GQD) is a classical TCM prescription originating from *Treatise on Cold Damage Diseases* (*Shang Han Lun*) by Zhang Zhongjing. It is composed of the plants *Pueraria lobata* (Willd.) Ohwi [Fabaceae], *Scutellaria baicalensis* Georgi [Lamiaceae], *Coptis chinensis* (Franch.) [Ranunculaceae], and *Glycyrrhiza uralensis* Fisch. [Fabaceae]. Intervention with different concentrations of GQD in ApoE^−/−^ AS mice revealed a concentration-dependent reduction in intraplaque deposits and foam cell accumulation. Protein expression levels of IL-1β, IL-18, ASC, GSDMD-N, GSDMD-C, NLRP3, pro-caspase-1, and NF-κB p65 were all downregulated to varying degrees, whereas the expression of CD206—a marker of M2 macrophages—was elevated (Zheng Y. et al., 2022). These findings suggest that GQD enhances plaque stability by modulating the NF-κB/NLRP3/caspase-1 signaling pathway. Specifically, it inhibits M1 macrophage polarization while promoting the M2 phenotype, suppresses the assembly and activation of inflammasome complexes, and alleviates macrophage pyroptosis.

#### 5.2.2 Simiao Yong’an decoction

Simiao Yong’an Decoction (SYD) is a classical formula originating from the *New Compilation of Proven Recipes* (*Yanfang Xinbian*), consisting primarily of the plants *Lonicera japonica* Thunb. [Caprifoliaceae], *Scrophularia ningpoensis* Hemsl. [Scrophulariaceae], *Angelica sinensis* (Oliv.) Diels [Apiaceae], and *Glycyrrhiza uralensis* Fisch. [Fabaceae]. SYD has been shown to improve blood lipid profiles and reduce hepatic lipid accumulation in ApoE^−/−^ AS mice. Mechanistically, it dose-dependently suppresses the protein expression levels of TLR4, NF-κB, NLRP3, caspase-1, IL-1β, and GSDMD. These findings suggest that SYD attenuates AS progression by inhibiting the TLR4/NLRP3/caspase-1 signaling pathway, thereby reducing pyroptosis activation (Yu N. et al., 2021).

#### 5.2.3 Jianpi Qutan Huayu decoction

Jianpi Qutan Huayu Decoction (JQHD) is a modified traditional Chinese prescription, derived from the classical *Sijunzi Decoction* combined with *Gualou Xiebai Banxia Decoction*, with the addition of *Prunus persica* (L.) Batsch [Rosaceae] and *Carthamus tinctorius* L. [Asteraceae]. JQHD significantly reduces blood lipid levels and aortic lipid deposition in ApoE^−/−^ AS mice. It also downregulates the expression of pyroptosis-related proteins, including NLRP3, caspase-1, and GSDMD. In an *in vitro* model of HUVEC injury, JQHD appears to inhibit endothelial pyroptosis by modulating the NLRP3/caspase-1/GSDMD signaling pathway, thereby improving endothelial cell viability and attenuating the initiation and progression of AS (Zhao et al., 2024).

#### 5.2.4 Xinmai Kang prescription

Xinmai Kang Prescription (XMKP) is composed of the plants *Imperata cylindrica* (L.) Raeusch. [Poaceae], *Curcuma zedoaria* (Christm.) Rosc. [Zingiberaceae], *Trionyx sinensis* (Wiegm.) [Trionychidae], *Citrus aurantium* L. [Rutaceae], *Aristolochia contorta* Bunge [Aristolochiaceae], and *Dendrobium officinale* Kimura & Migo [Orchidaceae]. XMKF has been shown to significantly reduce intimal thickness, necrotic cell burden, and pyroptosis in the arteries of ApoE^−/−^ AS mice. It markedly suppresses the expression of key inflammatory and pyroptosis-related markers, including ICAM-1, IL-18, VCAM-1, MMP-9, NLRP3, monocyte chemoattractant protein-1 (MCP-1), TLR4, caspase-1, IL-1β, and GSDMD-N (He et al., 2025; Xu et al., 2025). These findings suggest that XMKP may inhibit macrophage pyroptosis by modulating NLRP3 inflammasome activation, thereby suppressing inflammatory cell activity, reducing proinflammatory cytokine release, stabilizing vulnerable plaques, and enhancing overall plaque stability in AS.

#### 5.2.5 Vascular softening pill

Vascular softening Pill (VSP) is a modified formulation based on *Baohe Pill* from the traditional Chinese medical text *Danxi’s Heart Method*. It is composed ofthe plants *Crataegus pinnatifida* Bunge [Rosaceae], *Citrus reticulata* Blanco [Rutaceae], *Pinellia ternata* (Thunb.) Breit. [Araceae], *Astragalus membranaceus* (Fisch.) Bunge [Fabaceae], *Sal*via *miltiorrhiza* Bunge [Lamiaceae], *Panax notoginseng* (Burk.) F.H. Chen [Araliaceae], and *Raphanus sativus* L. [Brassicaceae]. VSP improves serum lipid profiles and alleviates pathological changes in aortic tissues and cells in ApoE^−/−^ AS mice. Mechanistically, it enhances phosphorylated AMP-activated protein kinase (P-AMPK) expression and suppresses the expression of NLRP3, cleaved caspase-1, GSDMD-N, IL-1β, and IL-18. These findings indicate thatVSP may exert its protective effects against AS by attenuating pyroptosis through modulation of the AMPK/NLRP3 signaling pathway (Qin H. et al., 2024).

#### 5.2.6 Zhuyu pill

Zhuyu Pill is a traditional Chinese herbal formula composed of *Evodia rutaecarpa* (Juss.) Benth. [Rutaceae] and *Coptis chinensis* (Franch.) [Ranunculaceae]. In ApoE^−/−^ AS mice, Zhuyu Pill significantly downregulates both protein and mRNA expression levels of NLRP3, ASC, and caspase-1, while markedly reducing the expression of pro-inflammatory cytokines IL-18 and IL-1β. These findings indicate that Zhuyu Pill effectively inhibits NLRP3 inflammasome activation and suppresses pyroptosis. Furthermore, Zhuyu Pill significantly reducesROS levels and suppresses the protein and mRNA expression of thioredoxin (TRX), TXNIP, NLRP3, ASC, and caspase-1. This suggests that its anti-pyroptotic and endothelial-protective effects may be mediated through modulation of the TMAO-activated ROS/TXNIP/NLRP3 signaling pathway and the downstream inflammatory cascade, thereby alleviating ECs injury and pyroptosis (Song et al., 2024).

#### 5.2.7 Qingxin Jieyu formula

Qingxin Jieyu Formula (QXJYF) is primarily composed of the plants *Astragalus membranaceus* (Fisch.) Bunge [Fabaceae], *Sal*via *miltiorrhiza* Bunge [Lamiaceae], *Ligusticum chuanxiong* Hort. [Apiaceae], *Pogostemon cablin* (Blanco) Benth. [Lamiaceae], and *Coptis chinensis* (Franch.) [Ranunculaceae]. Studies have shown that QXJYF can stabilize atherosclerotic vulnerable plaques through immunomodulatory mechanisms. In ApoE^−/−^ mouse models, this formula significantly reduces blood lipid levels and serum inflammatory cytokines, while also inhibiting macrophage pyroptosis mediated by the NLRP3 inflammasome. Further mechanistic investigations revealed that QXJYF activates the Sirt1/peroxisome proliferator-activated receptor gamma PPARγ/NF-κB signaling pathway, promoting the polarization of macrophages from the pro-inflammatory M1 phenotype to the anti-inflammatory M2 phenotype. Consistent results observed in both *in vivo* and *in vitro* studies suggest its strong anti-inflammatory and plaque-stabilizing potential (Song L., 2024).

#### 5.2.8 Zuogui Jiangtang Shuxin formula

Zuogui Jiangtang Shuxin Formula (ZGJTSXF) is a traditional Chinese herbal prescription composed of the plants *Astragalus membranaceus* (Fisch.) Bunge [Fabaceae], *Rehmannia glutinosa* (Gaertn.) DC. [Orobanchaceae], *Coptis chinensis* (Franch.) [Ranunculaceae], *Sal*via *miltiorrhiza* Bunge [Lamiaceae], *Cornus officinalis* Siebold & Zucc. [Cornaceae], *Crataegus pinnatifida* Bunge [Rosaceae], and *Pueraria lobata* (Willd.) Ohwi [Fabaceae]. Pharmacological studies have demonstrated that drug-containing plasma from ZGJTSXF significantly reduces the rate of cell apoptosis and inhibits LDH release, thereby preserving membrane integrity. Additionally, ZGJTSXF downregulates the expression of key pyroptosis-related proteins—NLRP3, caspase-1, and GSDMD—as well as apoptosis-related markers such as caspase-3 and Bax, thereby suppressing ox-LDL–induced macrophage pyroptosis (Yang et al., 2021). Further investigations indicate that ZGJTSXF protects macrophage function by modulating the TLR4/NF-κB/NLRP3 inflammatory signaling pathway and regulating the expression of pyroptosis-associated proteins. It effectively inhibits LPS-induced macrophage inflammatory injury and pyroptosis, contributing to the maintenance of macrophage homeostasis (Yang et al., 2022).

#### 5.2.9 Zhilong Huoxue Tongyu capsule

Zhilong Huoxue Tongyu Capsule (ZLHXTY) is a traditional Chinese medicinal formula composed of *Astragalus membranaceus* (Fisch.) Bunge [Fabaceae], *Lumbricus rubellus* Hoffmeister [Lumbricidae], *Tacca palmata* Forst [Dioscoreaceae], *Cinnamomum cassia* (L.). J. Presl [Lauraceae], and *Hirudo nipponia* (Hirudinidae). ZLHXTY exerts its anti-pyroptotic and cytoprotective effects by interfering with the recruitment and assembly of the adaptor protein ASC and pro-caspase-1. This inhibition suppresses the caspase-1–mediated cleavage of GSDMD and pro–IL-1β, thereby reducing the protein expression of their active forms—GSDMD-N and mature IL-1β. As a result, ZLHXTY mitigates membrane pore formation and proinflammatory cytokine release, ultimately inhibiting pyroptosis and promoting anti-inflammatory protection (Liu M. et al., 2020). Further studies have demonstrated that ZLHXTY significantly improves blood lipid profiles and reduces carotid plaque formation in a rabbit model of hyperlipidemia-induced carotid AS. The underlying mechanism is thought to involve the suppression of NF-κB phosphorylation, thereby inhibiting NLRP3 inflammasome activation and the release of downstream inflammatory mediators, contributing to its atheroprotective effects (Liu M. et al., 2021).

#### 5.2.10 Soufeng Qutan decoction

Soufeng Qutan Decoction is a traditional Chinese herbal formulation composed of *Buthus martensii* Karsch [Buthidae], *Scolopendra subspinipes* (Linnaeus) [Scolopendridae], *Lumbricus rubellus* Hoffmeister [Lumbricidae], *Hirudo nipponia* (Hirudinidae), *Citrus reticulata* Blanco [Rutaceae], *Pinellia ternata* (Thunb.) Breit. [Araceae], and *Atractylodes macrocephala* Koidz. [Asteraceae]. This decoction has been shown to significantly reduce the number of foam cells in the arterial intima and decrease atherosclerotic plaque area in ApoE^−/−^ mice. Mechanistically, it downregulates the expression of NLRP3, caspase-1, and ASC, thereby suppressing the assembly and activation of the NLRP3 inflammasome. Consequently, the secretion of downstream proinflammatory cytokines, such as IL-1β and IL-18, is markedly reduced (Zhao et al., 2022). By targeting the upstream events of the inflammatory cascade, Soufeng Qutan herbal medicine may exert its anti-atherosclerotic effects through modulation of immune-inflammatory pathways and inhibition of pyroptosis.

Current evidence from studies on TCM formulas suggests that one of their potential mechanisms in preventing and treating AS is the inhibition of pyroptosis. Most studies established relatively standardized control groups and provided detailed documentation of treatment protocols, which to some extent ensures the authenticity and reliability of the data. Nevertheless, several notable limitations remain. First, no study to date has combined TCM interventions with genetic manipulation in animal models, such as knockdown or knockout of key components of the NLRP3 inflammasome or pyroptosis-related inflammatory mediators, to directly verify the causal role of pyroptosis in the therapeutic effects of these formulas. Second, with the exception of the Vascular Softening Pill, other studies did not incorporate the use of specific inhibitors targeting the pyroptosis signaling pathway for mechanistic validation, thereby weakening the causal link between the suppression of pyroptosis and the amelioration of AS. *In vivo* experiments have mostly focused on structural improvements, with a lack of functional hemodynamic assessments. Future research should adopt multidimensional validation strategies, including genetically modified models, targeted pathway inhibition, and multi-omics analyses, to delineate the precise molecular targets of TCM formulas within pyroptosis and its upstream and downstream signaling networks, and to clarify their direct contributions and synergistic effects in AS prevention and treatment.

## 6 Therapeutic potential and challenges

### 6.1 Therapeutic potential

In recent years, a large number of basic and preclinical studies have shown that natural products have significant potential in regulating pyroptosis for the prevention and treatment of AS. Unlike single-target drugs, natural products typically exert multi-target and multi-pathway effects, intervening in multiple pathological processes such as oxidative stress, inflammation, mitochondrial dysfunction, and programmed cell death, thereby achieving pathological improvement at molecular, cellular, and tissue levels. For example, resveratrol, salvianolic acid B, and t scutellarin can inhibit the assembly and activation of the NLRP3 inflammasome, block the formation of cell membrane pores mediated by caspase-1/GSDMD, reduce the secretion of IL-1β and IL-18, and thereby reduce pyroptosis levels in macrophages and endothelial cells; salvianolic acid B, curcumin, and nobiletin improve ROS metabolic balance, inhibit oxidative stress-induced pyroptosis, and enhance redox homeostasis; Astragaloside IV not only inhibits the inflammasome but also promotes macrophage polarization from pro-inflammatory M1 type to anti-inflammatory M2 type via the STAT6/TGF-β pathway, improving plaque microenvironment. Clinical trials also indicate that TCM formulas (such as Gegen Qinlian Decoction, Zhilong Huoxue Tongyu Capsule) can significantly improve symptoms in patients with CHD, alleviate the degree of angina, reduce blood lipid levels, and improve quality of life (Tabanelli et al., 2021; Wang et al., 2025). Compared to single statins or anti-inflammatory drugs, natural products demonstrate unique advantages in long-term safety, systemic regulation, and multi-pathway intervention, making them a promising supplement or even a core strategy for the prevention and treatment of AS.

### 6.2 Challenges and limitations

Despite the promising prospects, the clinical translation of natural products in regulating pyroptosis for treating AS still faces multiple challenges: (1) Many active ingredients (such as resveratrol and curcumin) have issues like low solubility, poor oral bioavailability, and rapid first-pass metabolism, which make it difficult to maintain effective drug concentrations in the body (Tabanelli et al., 2021; Wang et al., 2025). (2) TCM formulas, composed of multiple herbs, face issues like variability in origin, processing methods, and component content, which affect the stability of their efficacy. Establishing component fingerprint maps and standardized extraction processes is key for future quality control. (3) Current research mostly focuses on the NLRP3 inflammasome pathway, but the pyroptosis regulatory mechanisms in different cell types (endothelial cells, smooth muscle cells, macrophages) may differ. Moreover, precise targeting of upstream triggers (such as cholesterol crystals, oxidized LDL, and mitochondrial damage) still requires further research. (4) Most evidence currently comes from animal models or *in vitro* studies, lacking multi-center, large-sample, randomized controlled clinical trials, which limits the formation of evidence-based recommendations. (5) AS is a chronic disease that requires long-term medication; therefore, the long-term safety and adherence to natural products still need large-scale clinical verification.

## 7 Conclusion and future directions

Pyroptosis plays a key role in the development and progression of AS. Natural products provide a promising approach for treating AS, offering various bioactive compounds with anti-inflammatory and cell-protective properties. A large number of studies have demonstrated the positive pharmacological and therapeutic effects of natural products on AS. However, current research is limited by specific practical issues, and proactive measures are required to promote the future use of natural products in clinical settings.

Currently, most studies on the effects of natural products on AS are at the signaling pathway level, lacking systematic analysis of the direct targets of natural products and their binding modes. Future research should focus on clarifying the molecular interactions between these natural compounds and key components of pyroptosis. Multi-omics analyses (such as transcriptomics, proteomics, and metabolomics), molecular docking, and molecular dynamics simulations can be used to predict and screen potential targets, while biological probes and active group probes combined with mass spectrometry can identify key binding proteins. Despite the substantial experimental evidence supporting the feasibility of using natural products to prevent and treat AS by inhibiting pyroptosis, the lack of high-level clinical evidence remains a major bottleneck for their promotion. In the future, standardized formulas or extracts should be developed to ensure consistency in clinical treatment effects and safety. Rigorous randomized, blind, or placebo-controlled single-center, small-scale exploratory studies should be conducted, gradually progressing to multi-center, large-sample, randomized, double-blind, placebo-controlled trials, to systematically evaluate the efficacy and safety of active monomers and TCM formulas in stabilizing plaques, reducing major adverse cardiovascular events, and plaque regression. To address the issues of low bioavailability and insufficient targeting, technologies such as nanoparticles, targeted liposomes, solid dispersions, and colloidal drug delivery systems can be developed for precise drug delivery to arterial plaques (Bhalani et al., 2022; Mahmood et al., 2023). At the same time, for personalized treatment, combining patients’ inflammatory profiles and genetic polymorphisms, the synergistic effects of natural products with conventional drugs (such as statins and antiplatelet agents) should be explored to construct comprehensive interventions that target multiple pathways and processes.

In conclusion, the importance of pyroptosis in the occurrence and progression of AS cannot be overstated. Its key role in driving vascular inflammation, endothelial damage, and plaque instability presents both challenges and new opportunities for disease prevention and treatment. Natural products, as a cutting-edge approach for exploring new therapies for AS, show immense potential due to their multi-target regulation of pyroptosis, improvement of the vascular microenvironment, and stabilization of atherosclerotic plaques. Future research should continue to elucidate their molecular mechanisms and therapeutic targets, and through systematic clinical validation, clarify their safety and efficacy, which is crucial for fully unlocking the potential of natural products in the prevention and treatment of AS.

## Glossary

ACD: accidental cell death
ACS: acute coronary syndrome
AhR: aryl hydrocarbon receptor
AIM2: activation of the absent in melanoma 2
APN: adiponectin
Arg-1: arginase-1
AS: atherosclerosis
ASC: apoptosis-associated speck-like protein containing
BAX: BCL2-associated X protein
BCL-2: B-cell lymphoma-2
CAR: chimeric antigen receptor
CARD: caspase recruitment domai
CD36: cluster of differentiation 36
CT: C-terminal domain
CTLs: cytotoxic T lymphocytes
CTSB: cathepsin B
CVD: cardiovascular disease
DAMPs: damage-associated molecular patterns
dsDNA: double-stranded DNA
ECM: extracellular matrix
ECs: endothelial cells
ELANE: elastase
ELF3: E74-like factor 3
eNOS: endothelial nitric oxide synthase
FGF21: Fibroblast growth factor 21
Gal-3: galectin-3
GAS: group A *streptococcus*
GSDMD: gasdermin D
GSDMD-NT: N-terminal fragment
GzmA: granzyme A
GZMB: granzyme B
H3K27me3: H3 lysine 27
HFD: high-fat diet
HIF-1α: hypoxia-inducible factor-1 alpha
HO-1: heme oxygenase-1
HSP70: heat shock protein 70
HUVECs: human umbilical vein endothelial cells
ICAM-1: intercellular adhesion molecule-1
IL-18: interleukin-18
IL-1β: interleukin-1β
LDH: lactate dehydrogenase
LDL: low-density lipoprotein
LPC: lysophosphatidylcholine
LPS: lipopolysaccharide
LSS: low shear stress
M1: pro-inflammatory
M2: anti-inflammatory
m6A: N6-methyladenosine
MCP-1: monocyte chemoattractant protein-1
miRNAs: microRNAs
MMPs: matrix metalloproteinases
mtROS: mitochondrial reactive oxygen species
NCCD: nomenclature committee on cell death
NEK7: NIMA-related kinase 7
NF-κB: nuclear factor kappa B
NINJ1: Ninjurin-1
NIX: NIP3-like protein X
NK: natural killer
NLR: NOD-like receptor
Nps: nanoplastics
NQO-1: NADPH quinone oxidoreductase-1
Nrf2: nuclear factor erythroid 2–related factor 2
NT: N-terminal domain
OS: oxidative stress
oxLDL: oxidized low-density lipoprotein
oxPAPC: oxidized phosphatidylcholine
PA: palmitic acid
P-AMPK: phosphorylated AMP-activated protein kinase
PAMPs: pathogen-associated molecular patterns
PCBs: polychlorinated biphenyls
PCSK9: proprotein convertase subtilisin/kexin type 9
PD-L1: programmed death-ligand 1
Pg: Porphyromonas gingivalis
pro-IL-1β: the precursors of interleukin-1β
PRRs: pattern recognition receptors
PYD: pyrin domains
RCD: regulated cell death
RIPK3: protein kinase 3
ROS: reactive oxygen species
SDHB: succinate dehydrogenase subunit B
STAT3: signal transducer and activator of transcription 3
T3SS: type III secretion system
TCM: traditional Chinese medicine
TET2: translocation methylcytosine dioxygenase 2
TGF-β: transforming growth factor-β
TLR2: Toll-like receptor 2
TMAO: Trimethylamine-N-oxide
TNF-α: tumor necrosis factor-α
TRX: thioredoxin
TXNIP: thioredoxin-interacting protein
UQCRC1: ubiquinol-cytochrome c reductase core protein I
VCAM-1: vascular cell adhesion molecule-1
VSMC: Vascular smooth muscle cell
ZBP1: Z-DNA binding protein 1
